# Genome-wide transcriptional landscape of *Mycobacterium tuberculosis* during acute lung infection

**DOI:** 10.3389/fimmu.2026.1875227

**Published:** 2026-08-28

**Authors:** Gunapati Bhargavi, Saleena Ghanny, Patricia Soteropoulous, Umashankar Vetrivel, Roja Jayaraman, Selvakumar Subbian

**Affiliations:** 1 Public Health Research Institute, Rutgers-New Jersey Medical School, Newark, NJ, United States; 2 The Genomics Center, Rutgers-New Jersey Medical School, Newark, NJ, United States; 3 Department of Virology and Biotechnology, Bioinformatics Division, Indian Council for Medical Research -National Institute for Research in Tuberculosis (ICMR-NIRT), Chennai, India; 4 Department of Medical Sciences, Academy of Scientific and Innovative Research (AcSIR), Ghaziabad, Uttar Pradesh, India

**Keywords:** cell wall, disease pathology, KEGG pathway, lung tuberculosis, mycobacterial adaptation, networks, pathways, rabbit Mtb infection

## Abstract

Tuberculosis (TB) remains a major global health burden, yet the mechanisms by which *Mycobacterium tuberculosis* (Mtb) adapts to host environments to drive disease pathology are incompletely defined. A key limitation has been reliance on axenic culture systems that fail to recapitulate the complex, host-imposed stresses encountered by Mtb *in vivo*. Here, we report the first microarray-based genome-wide transcriptomic profiling of Mtb in rabbit lungs with active TB, which closely mirrors human disease features, including granuloma heterogeneity, necrosis, and cavitation. Using Mtb RNA isolated from infected lung homogenates or broth-culture, we capture bacterial transcriptional states shaped by the host microenvironments. The transcriptional data analyses reveal extensive, context-dependent reprogramming of Mtb metabolic, respiratory, and stress-response networks that diverges markedly from *in vitro* expression profiles, including activation of stress adaptation, lipid catabolism, nucleic acid metabolism, and transcriptional regulation pathways. These data uncover pathways and networks that are selectively engaged *in vivo* and likely critical for Mtb survival within granulomatous lesions. Our findings demonstrate that transcriptional states most relevant to TB pathogenesis are underrepresented in standard lab-grown Mtb models and highlight the importance of *in vivo* bacterial profiling. By characterizing Mtb gene expression within diseased lungs, this study provides a systems-level framework for understanding TB pathogenesis and reveals *in vivo*-essential pathways, offering potential targets for translational drug discovery and the development of more effective anti-TB therapies.

## 1 Introduction

Tuberculosis (TB) remains the number one cause of death among infectious diseases, with an estimated 10.7 million new cases and approximately 1.23 million deaths in 2024 (1). These numbers highlight the persistent gap between the global TB burden and the World Health Organization’s vision of a “TB-free world” by 2025 (1). *Mycobacterium tuberculosis* (Mtb), the causative agent of TB, primarily infects the lungs, where alveolar macrophages phagocytose inhaled bacilli, triggering immune cell recruitment and granuloma formation (2, 3). Although granulomas can restrict bacterial dissemination, Mtb can survive and replicate within these lesions, enabling disease progression (2–4). However, the mechanistic understanding of how Mtb adapts to and thrives within the lungs remains incomplete (5).

Mtb transcriptional programs in infected lungs are distinctly shaped by host-imposed conditions that are poorly captured in bacteria grown in broth cultures (2, 6). Within the granulomas, Mtb encounters fluctuating oxygen tension, reactive oxygen and nitrogen species (ROS and RNS), acidic and nutrient-limited compartments. These stresses drive extensive reprogramming of Mtb physiology and stress responses, which may not be observed in broth-grown bacteria (7, 8). For example, studies from *in vivo* and ex vivo transcriptional profiling show that Mtb induces iron acquisition pathways, alternative respiratory systems, and dormancy-associated regulons that are attenuated or qualitatively distinct in broth-grown bacilli (7, 8). In mouse lungs, Mtb shifts away from conventional oxidative phosphorylation and redirects carbon flux toward glycolysis, consistent with granulomatous metabolic constraints (9, 10). Similarly, RNA-seq analyses of sputum from patients with pulmonary TB reveal enrichment of Mtb transcripts associated with lipid catabolism, stress adaptation, and alternative energy metabolism relative to *in vitro* culture (11). Moreover, dual RNA-seq studies and meta-analyses have identified a conserved *in vivo* transcriptional signature of Mtb, characterized by hypoxia response, lipid utilization, and redox regulation, underscoring the biological divergence between the *in vivo* and *in vitro* (lab-grown) transcriptomes of Mtb (12–14). Therefore, defining these *in vivo* transcriptional adaptations is critical for understanding TB pathogenesis and identifying novel therapeutic targets for effective anti-TB drug development (7, 12, 13).

Among clinically relevant Mtb strains, the hypervirulent Beijing lineage strain HN878 is of particular interest. We previously reported that HN878 infection in the rabbit model leads to the formation of cavitary and necrotic granulomas through induction of inflammation and host cell death (15). Although Mtb HN878-infected rabbits recapitulate key pathological features of human TB, including granuloma heterogeneity, necrosis, and cavitation, a comprehensive transcriptomic analysis of Mtb in rabbit lungs has not been reported previously (15–19). Previous studies in this model have been limited to targeted qRT-PCR analyses of a small subset of Mtb genes, leaving the broader *in vivo* transcriptional landscape of Mtb in rabbit lungs largely unexplored. In the current study, we present the first microarray-based genome-wide analysis of the Mtb transcriptome during progressive pulmonary TB in rabbit lungs, revealing regulatory pathways associated with context-dependent bacterial adaptations across distinct lung microenvironments (15–17, 20, 21). We postulate that bacilli isolated during the acute stage of infection (four weeks post-infection) represents a transcriptional state optimized for survival and growth under host immune pressure, rather than for dormancy, which are more characteristic of chronic stages of infection.

## 2 Results

### 2.1 Pulmonary granuloma architecture and bacterial burden in rabbit lungs with acute TB

**Figure 1:**
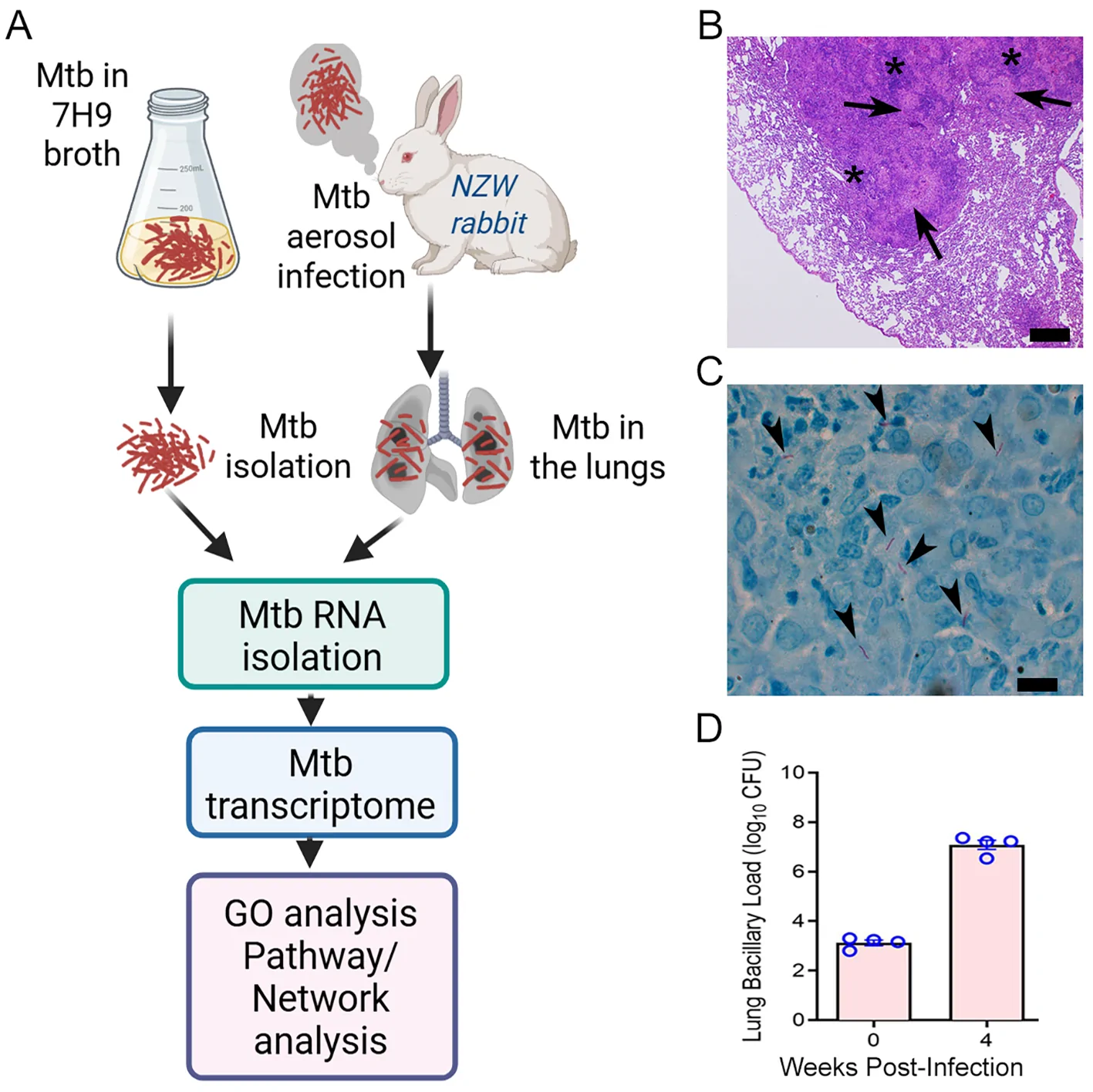
Experimental scheme, lung pathology, and bacterial load in Mtb-infected rabbits. **(A)** RNA was isolated from Mtb grown in broth or from infected rabbit lungs (n=4) and used for microarray gene expression analysis. The transcriptome profile of Mtb in the lungs, relative to broth culture, was further subjected to gene ontology (GO), pathway, and network analysis. **(B)** H&E-stained lung section of Mtb-infected rabbits at 4 weeks (acute infection). Large, coalescent granulomas with signs of early necrosis (arrows) and lymphocyte accumulation (asterisks) were noted in the acute stage of infection. Image taken at 100x magnification, and the scale bar is 100 microns. **(C)** AFB-stained rabbit lung section with acute Mtb infection showing copious bacterial presence (arrow heads). Image taken at 1000x magnification, and the scale bar is 10 microns. **(D)** Bacterial CFU in Mtb-infected rabbit lungs at 4 weeks. Values plotted are mean log_10_ CFU +/- standard deviation (error bars) from n=4 rabbits per time point.

As shown in the experimental design schema (**Figure 1A**), the differential Mtb gene expression in rabbit lungs during the acute stage of infection was compared to Mtb grown in broth culture. The histopathological assessment of rabbit lungs at four weeks post-infection revealed well-organized solid granulomas characterized by dense cellular infiltrates and areas of tissue consolidation with minimal necrosis (**Figure 1B**) (18). Ziehl–Neelsen staining revealed abundant Mtb in the granulomatous regions (**Figure 1C**), indicating that the bacteria persist within this inflammatory milieu. The lesions are morphologically analogous to early-to-mid-stage human TB granulomas that are known to impose gradients of oxygen, nutrients, and immune effector molecules to Mtb (4, 18, 22). Consistent with the disease pathology, the rabbit lung bacillary loads increased markedly at four weeks post-infection, reaching ~7 log_10_ CFU, compared to the baseline (T = 0) (**Figure 1D**). This growth phase precedes effective immune containment by the adaptive immunity and reflects a window during which Mtb must balance replication with adaptation to escalating host stressors (18, 23, 24).

### 2.2 Overview of Mtb transcription profile in rabbit lungs

**Figure 2:**
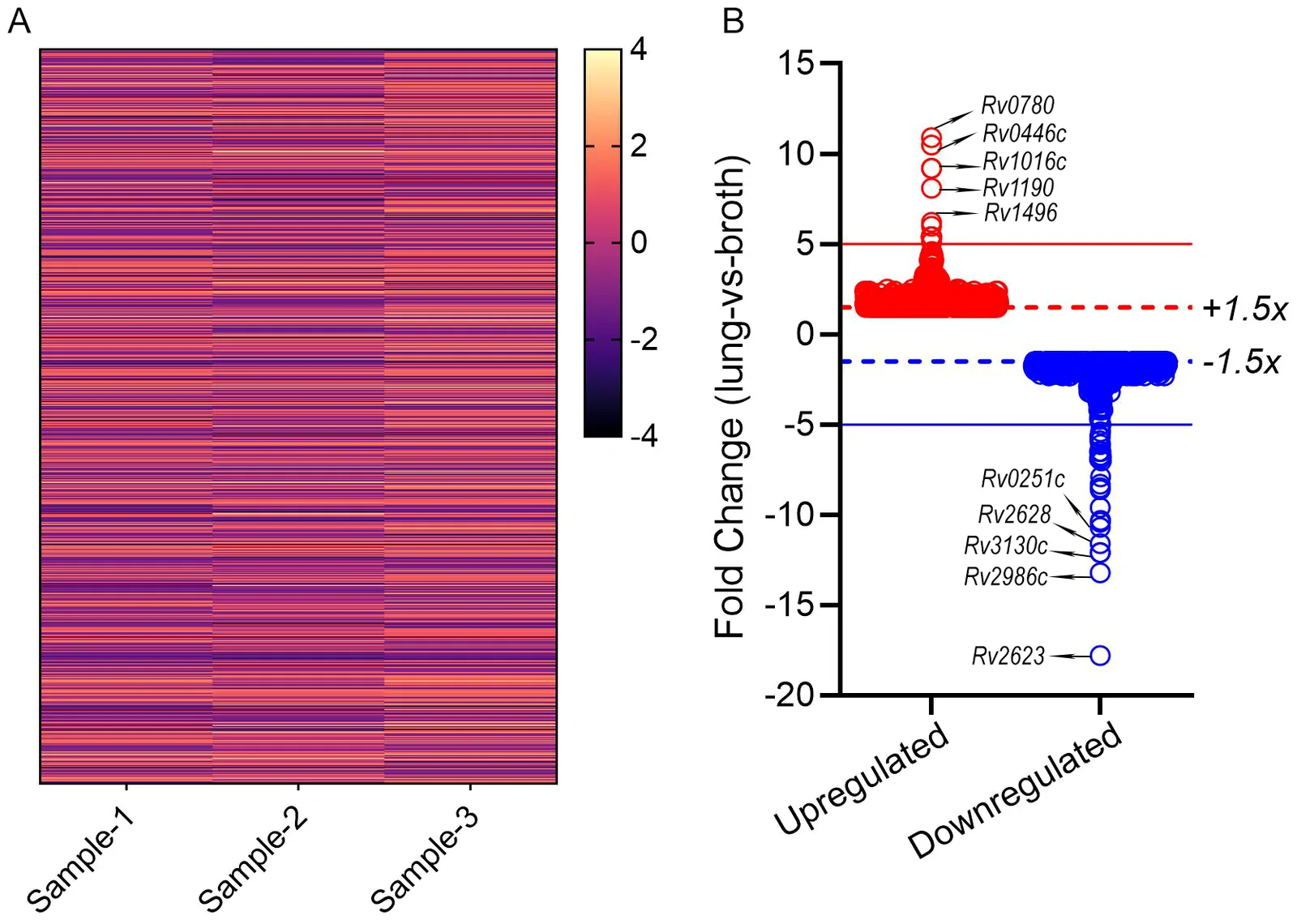
Overview of Mtb transcriptome during acute infection in rabbit lungs. **(A)** Heat map of Mtb transcriptome in the lungs of three rabbits (biological replicates). Each row represents a gene, and the red color indicates upregulation, and the blue color indicates downregulation, as shown in the scale bar. **(B)** Scatter plot of up (red color) and down (blue color) regulated genes based on a 1.5-fold cutoff. The top five up and down-regulated genes were indicated by corresponding gene IDs.

Unsupervised clustering of 4,089 genes revealed a consistent transcriptional profile across biological replicates, indicating robust and reproducible adaptation of Mtb to the *in vivo* environment (**Figure 2A**). Although some inter-sample variability in the magnitude of expression was observed, approximately 90% of transcripts exhibited consistent directions of regulation across all tested samples. This indicates that the dominant biological response was consistent and highly concordant among the samples. Therefore, averaging expression values across the three biological replicates was considered appropriate to capture the shared biological signals, while minimizing the impact of sample-specific variations. Of the 1,118 gene transcripts selected using a ±1.5-fold change threshold, 575 were upregulated, and 543 were downregulated in the lungs. Of these, 107 transcripts remained significantly altered even under a more stringent ±3-fold threshold (**Figure 2B**, **Supplementary Table 1**). The top upregulated genes include *Rv0708* (Ribosomal protein)*, Rv0446c* (Cell wall and cell processes)*, Rv1016c* (Conserved lipoprotein), *Rv1190* (hypothetical protein), and *Rv1496* (Kinase transporter), whereas the prominently downregulated genes include *Rv0251c* (Heat shock protein)*, Rv2628* (DosR-regulated gene associated with the hypoxic stress response), *Rv3130c* (a DosR-regulated triacylglycerol synthase involved in lipid storage during hypoxia and dormancy), *Rv2986c* (DNA binding protein), and *Rv2623* (a DosR-regulated Universal stress protein) (**Figure 2B**). Collectively, the transcriptional profile indicates a shift in Mtb biology in the lungs toward enhanced protein synthesis, cell envelope remodeling, and transport-associated functions, as evidenced by the upregulation of relevant genes in these processes. In contrast, the marked downregulation of heat shock and DosR-regulated genes suggests suppression of dormancy and persistence-associated programs. Overall, these findings are consistent with a physiological state of Mtb, favoring active growth and metabolic activity during acute infection, rather than hypoxia-induced dormancy.

. The microarray gene expression data was corroborated and validated using qPCR assay of randomly selected Mtb genes, to avoid any bias based on expression levels (**Table 1**). The qPCR results are consistent with the expression pattern and direction as observed in the microarray analysis.

**Table 1:** Validation of Mtb microarray data by qPCR.

*Genes*	FC in lungs (vs broth) by microarray		FC in lungs (vs broth) by qPCR	
	*Avg*	*sd*	*Avg*	*sd*
*narX*	-1.29585	0.050167	-1.49848	0.387348
*narK2*	-2.52159	0.552916	-1.28197	0.232745
*fadD26*	-1.10413	0.105763	-1.1795	0.337518
*hspX*	-4.79121	1.620361	-1.10586	0.109391
*mmpL8*	1.150942	0.095785	1.905635	2.497495
*sigF*	-1.74027	0.308387	-17.1757	35.68246
*sigH*	-1.2989	0.0496	1.186787	0.360079
*sigA*	1.73301	0.588582	-1.25894	0.445114
*bfrA*	2.00394	0.838751	1.327306	0.601991
*lipF*	-0.42365	1.249609	-1.29632	0.543045
*pckA*	-1.30733	0.151166	-1.09418	0.20543
*dnaE2*	-1.16594	0.065759	-1.15792	0.489352
*relA*	-1.08915	0.061029	-1.61629	1.14992
*katG*	-2.2348	0.584874	-1.14739	0.318232
*ahpC*	-1.10754	0.080498	-1.16322	0.347963
*kasA*	-2.05709	0.522657	-1.65745	1.756929
*efpA*	1.371704	0.219111	1.036889	0.077361
*dfrA*	1.046801	0.030532	1.039075	0.097967
*sodA*	1.137932	0.0904	1.037609	0.06274
*sodC*	1.151923	0.100857	1.133697	0.214626
*fbpB*	-1.0258	0.036888	-1.08654	0.181772
*whiB1*	-4.90302	4.305243	-1.02028	0.027483
*murD*	-1.2824	0.122758	-1.37682	0.736858
*ftsQ*	1.173754	0.006502	1.024094	0.07991
*Rv0283*	-1.11325	0.115234	-1.02548	0.056697

### 2.3 Biological processes affected by differential Mtb gene expression

**Figure 3:**
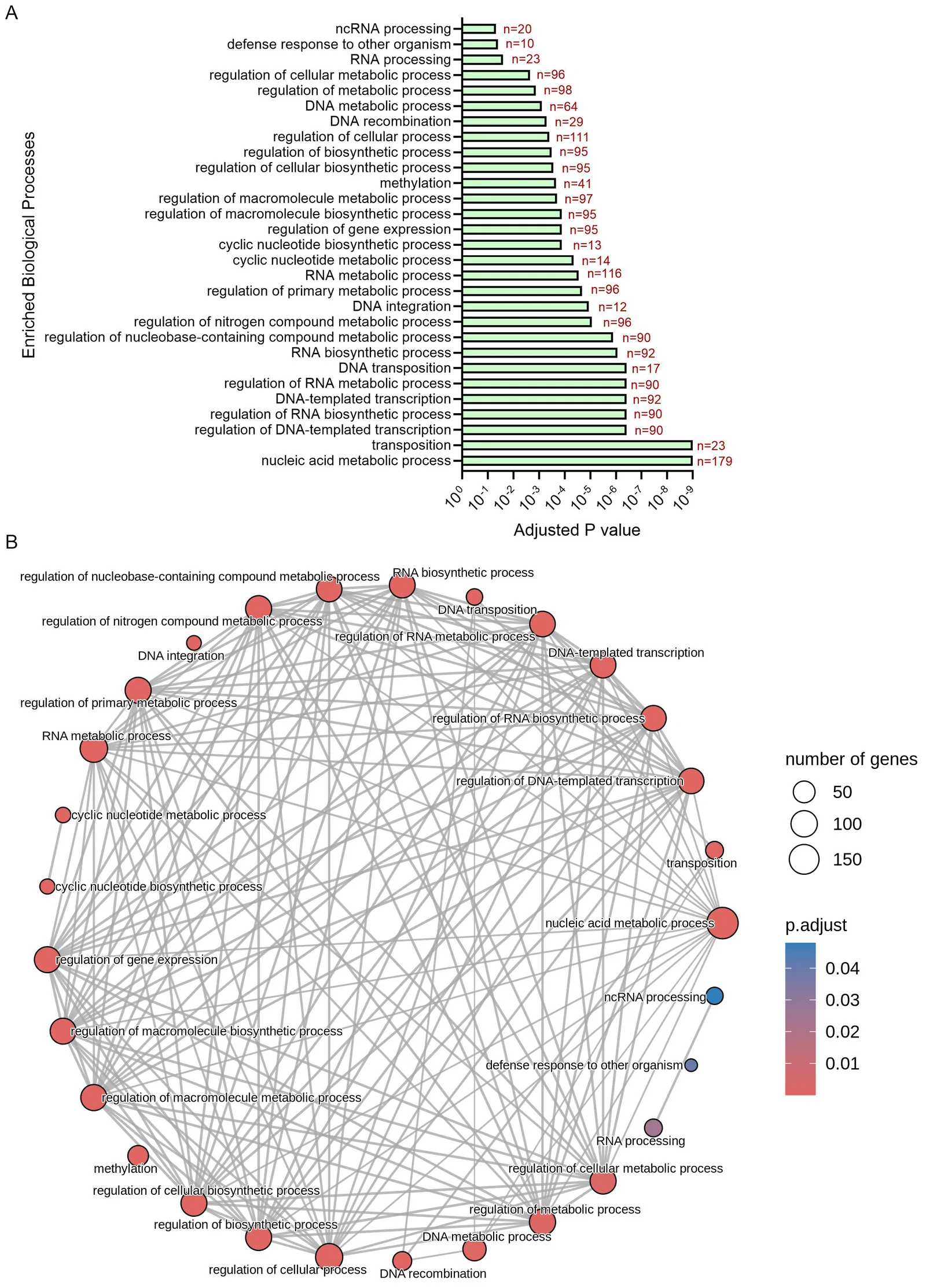
Key biological processes of Mtb were altered in rabbit lungs. **(A)** Bar graph showing significantly affected biological processes of Mtb in the lungs, compared to the broth cultures. The x-axis denotes the adjusted p-value of significance, and the y-axis shows enriched biological processes. The number of Mtb genes involved in each of the biological processes is indicated next to the corresponding bars. **(B)** Interaction map showing the link between different biological functions as mentioned in **(A)**. The size of the circle is proportionate to the number of genes, and the color code is based on the p-value of significance.

Comparative transcriptomic analysis of Mtb isolated from rabbit lungs versus broth-grown cultures suggest extensive remodeling of biological processes that enable bacterial survival during infection (**Figure 3**, **Supplementary Table 2**). The GO enrichment analysis indicate that Mtb genes associated with nucleic acid metabolic process (q value: 7.4E-10), RNA biosynthesis (q value: 8.24E-07), RNA processing (q value: 0.02), and regulation of gene expression (q value: 0.0001) were significantly enriched, suggesting that Mtb actively reconfigures its transcriptional machinery to respond to the host environment (**Figure 3A**). Consistently, expression of the TetR family transcription factors such as *Rv0144*, a DNA-binding regulator previously linked to environmentally responsive regulons, were enriched. In addition, genes associated with ncRNA biology and RNA maturation pathways, including *Rv2630*, *Rv2554c*, *Rv2631*, and *Rv3421c*, were enriched (q value: 0.045). In parallel, genes involved in DNA metabolism and genome maintenance, including DNA recombination (q value: 0.0004), transposition (q value: 7.41E-10), and integration (q value: 1.05E-05), were also enriched, highlighting Mtb’s survival adaptations under host-induced stress conditions. Some of the key representative genes included SufB (Rv1461) that codes for Fe–S cluster assembly protein as well as multiple genes that codes for insertion sequence-associated transposases, suggesting activation of transcriptional programs involved in DNA repair, genome plasticity, and adaptive evolution (25) (**Figure 3A**).

Beyond nucleic acid-associated processes, lung-resident Mtb exhibited significant enrichment of genes governing metabolic regulation pathways. Particularly, Mtb genes associated with nitrogen compound metabolism (q value: 8.20E-06), primary metabolism (q value: 1.97E-05), nucleobase-containing compound metabolism (q value: 1.21E-06) and cyclic nucleotide metabolic processes (q value: 4.23E-05) were overrepresented, reflecting Mtb’s transcriptional adjustments in nutrient utilization and metabolic homeostasis. Notably, expression of transcriptional regulator Rv1358 that links metabolic adaptation with transcriptional regulation was upregulated (**Figure 3A**). Similarly, genes associated with methylation, including *Rv1498c*, *Rv0187*, *Rv2675c*, also formed a distinct enriched cluster in the transcriptome of lung-derived Mtb (q value: 0.0002), along with several methyltransferases involved in macromolecular modification and metabolic regulation of Mtb *in vivo*.

Importantly, the Mtb genes impacting biological processes identified *in vivo* extended beyond transcriptional control and metabolism. Induction of transcriptional programs linked to hypoxia response and redox homeostasis pointed to adaptation to the oxygen-limited and nitrosative conditions typical of granulomatous lesions (26). Additionally, a subset of Mtb genes associated with defense response to other organisms, including *Rv2816c*, *Rv2817c*, and *Rv2818c*, were specifically enriched in the lungs (q value: 0.037) (**Figure 3A**). Importantly, the biological processes impacted by the transcriptional changes are highly interconnected, linking nucleic acid metabolism, nitrogen regulation, oxidative and nitrosative stress defense, cell envelope remodeling, and metal ion homeostasis into an integrated adaptive program (**Figure 3B**). However, expressions of other dormancy/stress response genes, such as *narK2*, *hspX*, *sigF*, and *whiB1* were downregulated, suggesting the complexities in the regulation of Mtb gene expression *in vivo*. Collectively, these transcriptional responses represent well-established strategies that enable Mtb to withstand macrophage-derived antimicrobial stress and nutrient sequestration to persist within infected tissues (27, 28).

### 2.4 Molecular functions impacted by differentially regulated Mtb genes

**Figure 4:**
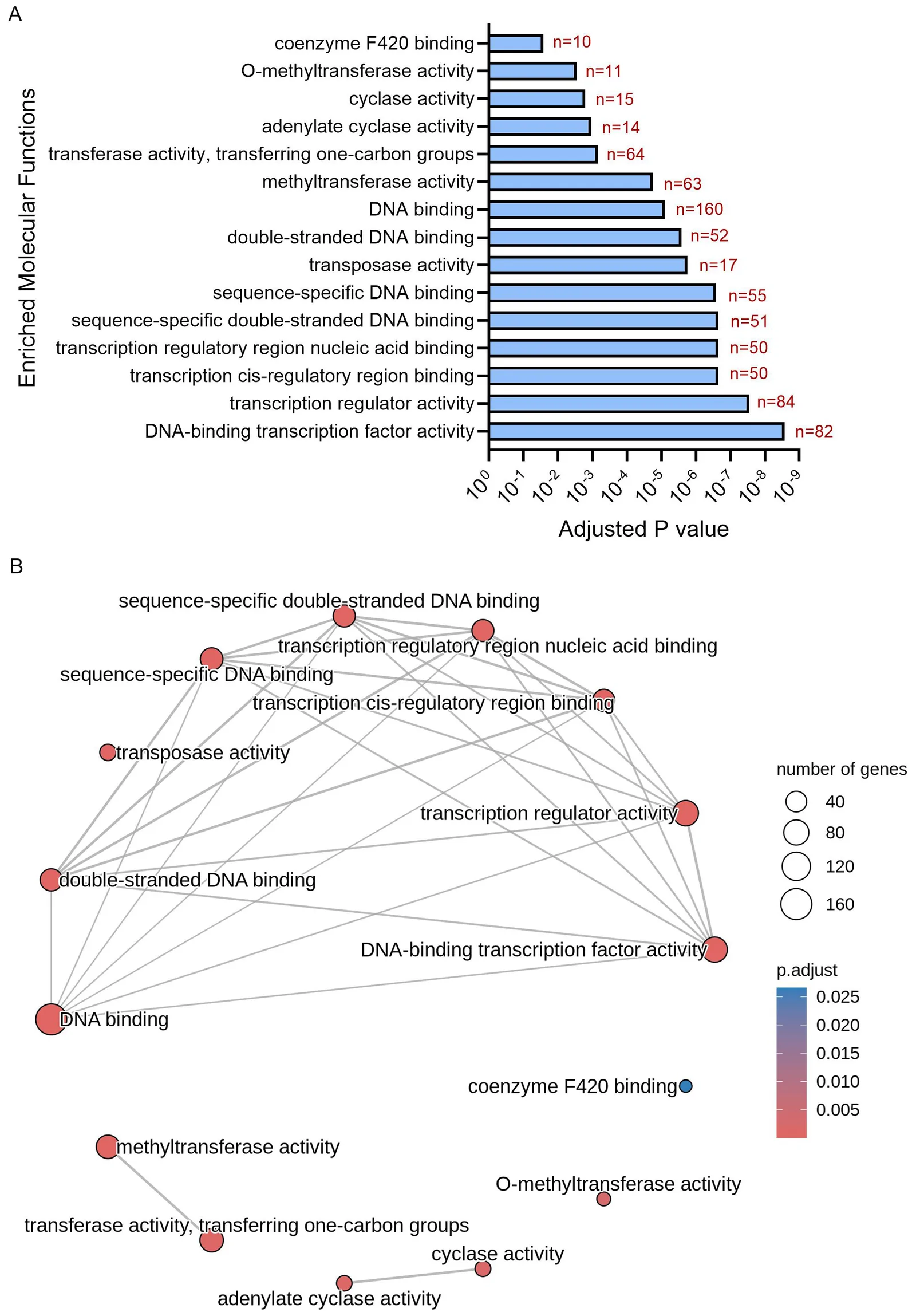
Key molecular functions of Mtb are altered in rabbit lungs. **(A)** Bar graph showing significantly affected molecular functions of Mtb in the lungs, compared to the broth cultures. The x-axis denotes the adjusted p-value of significance, and the y-axis shows enriched molecular functions. The number of Mtb genes involved in each of the molecular functions is indicated next to the corresponding bars. **(B)** Interaction map showing the link between different molecular functions as mentioned in **(A)**. The size of the circle is proportionate to the number of genes, and the color code is based on the p-value of significance. Note that some of the molecular functions are not linked to any other enriched functions.

The transcriptional profile enriched in lung-derived Mtb translates into a molecular functional landscape dominated by transcriptional regulation and DNA-associated activities (**Figure 4**; **Supplementary Table 3**). The most significantly enriched molecular functions included DNA-binding transcription factor activity (q value: 2.63E-09), transcription regulator activity (q value: 2.88E-08), sequence-specific DNA binding (q value: 2.60E-07), and transcription regulator binding (q value: 2.27E-07), reinforcing the central role of gene regulatory networks during infection. Within this regulatory landscape, several genes encoding for global transcriptional regulators, including Fur, IscR, Lrp, and factors associated with the sigma factor SigC/RpoN-dependent pathways, were enriched. These proteins coordinate Mtb responses to iron limitation, Fe–S cluster homeostasis, amino acid metabolism, and nitrogen utilization (29–31).

The enrichment of Mtb transcripts associated with DNA metabolism pathways implies that the molecular functions related to genome protection were also significantly enriched in lung-derived Mtb (**Figure 4A**). These included sequence-specific double-stranded DNA binding (q value: 2.27E-07) and damaged DNA binding (q value: 8.05E-06). Enriched Mtb transcripts in these categories codes for homologs of HU family nucleoid-associated proteins, which maintain chromosomal architecture and protect DNA integrity (32), as well as LexA-like repressors that regulate the SOS DNA damage response (33). In addition, genes encoding methyltransferases (q value: 1.68E-05), including Dam-like DNA adenine methyltransferases involved in replication timing and transcriptional regulation (34, 35), and SAM-dependent small molecule methyltransferases (q value: 2.85E-03), encompassing enzymes linked to detoxification and metabolic flexibility (36, 37) were significantly enriched. Thus, the transcriptional profile suggests active epigenetic regulation and metabolic diversification in Mtb during lung infection.

In addition, transcriptional changes linked to cellular metabolism and redox homeostasis were enriched. Enrichment of transcripts linked to transferase activities involved in one-carbon metabolism (q value: 7.01E-04), GcvT-like glycine cleavage pathways, and FAD-dependent oxidoreductase functions (q value: 2.66E-02) suggest that Mtb actively adjusts carbon utilization and redox balance to accommodate fluctuating oxygen availability within lung lesions (38, 39).

Network analyses of differentially expressed Mtb genes revealed that most enriched molecular functions were interconnected and integrated with broader, *in vivo*-adaptive pathways identified at the biological-process level (**Figure 4B**). However, the expression profile of Mtb genes involved in coenzyme F420 binding, O-methyltransferase activity, and transposase activity appeared to operate more independently, indicating specialized roles during *in vivo* infection (**Figure 4B**). Overall, these findings suggest that lung-resident Mtb relies on coordinated transcriptional control, genome maintenance, epigenetic regulation, and redox adaptation to survive within the host.

Apart from the networks, individual genes involved in TB pathogenesis were also differentially regulated in the lungs. Expression of PPE family genes, including *Rv2123*, *Rv1039c* and *Rv3532*, as well as ESX-1 associated genes, such as *Rv3614c* were upregulated, while expression of some virulent-associated genes such as Esat6 (*Rv3874*), and *Rv2623* was downregulated. This pattern highlights Mtb’s complex virulence regulation and host-adaptation machinery *in vivo*.

### 2.5 Differentially regulated Mtb KEGG pathways

The biological and molecular adaptations, derived from the transcriptional landscape of Mtb in the lungs converged on specific metabolic pathways as revealed by KEGG analysis. Biosynthesis of secondary metabolites pathway, which includes polyketide synthases (PKSs), lipid-modifying enzymes, and accessory metabolic genes, was the most prominently enriched pathways (**Supplementary Figures 1**, **2**). Strong representation of genes such as *Rv2244*, *Rv2220*, *Rv1438*, and *Rv3810*, together with enriched PKS loci including *pks12*, *pks13*, and *ppsA* in this pathway points to enhanced production of virulence-associated lipids such as phthiocerol dimycocerosates (PDIMs), which promote immune evasion and intracellular survival (40, 41). Upregulation of *pks11* and *pks18* further suggests increased synthesis of specialized long-chain lipid metabolites that may contribute to adaptation within host tissues (42).

In contrast, components of the dTDP-L-rhamnose biosynthesis pathway (*rmlA–D*), required for cell wall arabinogalactan assembly (43), were downregulated. Similarly, multiple genes within the fatty acid synthesis II and mycolic acid biosynthesis pathways, including *kasA*, *kasB*, *accD6*, and *InhA*, were downregulated (**Supplementary Figure 2**). Genes involved in trehalose-containing lipid metabolism, such as the antigen 85 complex (*fbpA*, f*bpB*, f*bpC*) and associated acyl-CoA carboxylase components, were also suppressed. Despite this broader reduction in anabolic lipid synthesis, selected pathways involved in lipid remodeling and maintenance of cell envelope integrity remained active. Expression of genes encoding mycolic acid modification enzymes, including *desA1, desA2*, and several *mmaA***-**family methyltransferases, were upregulated or, unchanged, suggesting continued refinement of the cell envelope. These observations indicate that Mtb shifts away from energetically costly growth-associated biosynthetic programs while preserving lipid modification pathways essential for persistence and virulence.

## 3 Discussion

The transcriptional landscape of Mtb recovered from rabbit lungs reveals extensive host-driven reprogramming. Our data suggests that rather than entering a uniform dormant state, lung-resident Mtb maintained active transcription and translation while engaging selective stress responses and metabolic adaptation. This notion is also supported by the significant downregulation of several DosR-regulated genes, which indicates a more metabolically active, growth-associated physiological state. These findings are consistent with previous studies showing that host niches impose dominant constraints on Mtb physiology (13, 44, 45).

A prominent feature of Mtb transcriptome in the lungs was the enrichment of transcriptional regulators and RNA-associated processes. Increased expression of TetR-family regulators, global regulators such as Fur, IscR, and Lrp, and RpoN-associated networks suggest extensive regulatory rewiring that enables adaptation to change in nutrient availability, redox status, and immune-mediated stress (29, 46, 47). Enrichment of RNA-processing and ncRNA-related factors further indicates an increased reliance on post-transcriptional regulation during infection.

Upregulation of DNA repair pathways, SOS response regulators, HU-like nucleoid proteins, and Fe–S cluster biogenesis genes reflect substantial genomic stress to Mtb in the lung environment (48, 49). Perhaps, exposure to reactive oxygen and nitrogen species, acidic pH, and immune-mediated damage likely necessitates a heightened genome protection for Mtb. The enrichment of transposases and recombination-associated genes also suggests increased genomic plasticity that may promote adaptation under immune pressure in the lungs. Metabolic reprogramming was another key transcriptional feature of the lung-adapted Mtb. Enhanced nitrogen metabolism, cyclic nucleotide signaling, one-carbon metabolism, and redox-related pathways indicate a shift toward energy conservation, redox balance, and persistence of Mtb during nutrient limitation in the lungs (50). These adaptations are consistent with Mtb survival in heterogeneous granuloma environments with fluctuating oxygen and carbon availability (26).

The transcriptome also showed activation of methylation-related pathways, implicating both epigenetic regulation and methyl-group-dependent metabolism in host adaptation. Upregulation of Mtb genes of Dam-like DNA methyltransferases and SAM-dependent enzymes suggests their roles in gene regulation, replication, and stress adaptation under host-imposed stress (35, 51). Although some DosR-associated dormancy genes were induced, several canonical stress-response genes, including *hspX, narK2, sigF*, and *whiB1*, were downregulated, possibly reflecting the heterogeneity in the physiological state of Mtb subpopulations in rabbit lungs (16–18). Alternatively, the bacteria may not be undergoing an acute DosR induction phase. Instead, they may occupy a later or alternative hypoxic state characterized by maintenance of selected hypoxia-associated pathways, regulatory rewiring, or transition toward enduring persistence. It is also possible that the DosR regulon, hypoxia adaptation, and general stress responses may overlap but are not identical programs, and their expression can become uncoupled depending on the Mtb strain, the stage of adaptation and environmental context (52, 53).

Extensive remodeling of lipid metabolism was particularly evident from the lung-derived Mtb transcriptome. Enrichment of secondary metabolite biosynthetic pathways, including multiple PKS families, underscores the importance of lipid-derived virulence factors, such as phthiocerol dimycocerosate (PDIM), phenolic glycolipids, and α-pyrones, which are known to modulate host immune recognition and intracellular survival of Mtb (40, 41). Although expression of *pks12*, *pks13*, *ppsA*, and type III PKSs was upregulated, the concurrent downregulation of *fadD26* indicates a mixed regulatory state within the PDIM biosynthesis network, For example, PDIM-associated pathways are transcriptionally engaged but not fully activated, because the concurrent downregulation of fadD26 creates a rate-limiting block at an early substrate-activation step. Thus, the expression profile is more consistent with partial activation or remodeling of PDIM-related lipid metabolism rather than robust enhancement of PDIM synthesis and accumulation (54). Biologically, this could reflect a stress-adaptation state in which *M. tuberculosis* maintains expression of downstream PDIM machinery while limiting the metabolic cost of full PDIM production (55). At the same time, repression of growth-associated fatty acid elongation pathways, coupled with maintenance of selected mycolic acid modification enzymes, indicates a shift from biomass production toward qualitative remodeling of the cell envelope (56, 57). Such remodeling likely enhances envelope flexibility, immune evasion, and long-term persistence of Mtb in the lungs (58, 59). The expression pattern of virulence and immune evasion genes, including the PPE family, associated with antigenic variation and modulation of host immune responses, and ESX-1-associated protein implicated in phagosomal disruption and cytosolic access suggests that Mtb strategically modulates virulence mechanisms to promote immune evasion, persistence, and establishment of a long-term infection niche while minimizing excessive host immune activation.

Collectively, these findings support the view that Mtb’s adaptation during acute lung infection is driven by a dynamic transcriptional state that favors active replication and protection from host-imposed stress, rather than a linear trend towards dormancy. An important implication is that transcriptional states most relevant to persistence and disease may be absent or underrepresented in standard culture conditions. By profiling Mtb directly from infected lung tissue, this study captures bacterial responses within the host environment and highlights pathways that may be essential *in vivo* but dispensable *in vitro*. The results emphasize lipid metabolism, regulatory plasticity, and genome protection as core pillars of Mtb survival in the host and highlight infection-relevant pathways as priority targets for therapeutic intervention.

We acknowledge that the transcriptional analysis was performed with three biological replicates from a single infection state, which might underestimate the transcriptomic heterogeneity arising from factors such as differences in granuloma composition and bacterial population among animals. In addition, RNA extracted from whole lungs may likely obscures lesion-specific variation in Mtb transcription profile. Nevertheless, the strong clustering of biological replicates and consistent regulatory pattern of gene networks support the robustness of the findings. However, the transcriptomic data must be validated by further functional assays to imply and ensure their biological significance.

In summary, pulmonary infection in rabbits induces extensive network-level transcriptional reprogramming in Mtb, reflecting metabolic flexibility, stress resilience, and adaptation to host immune pressures. Integrating pathology, microbiology, and transcriptomics provides a systems-level view of TB pathogenesis. In this context, our analysis of Mtb transcriptional landscape in the lung advances understanding of the bacterial adaptation strategies that shape host-pathogen interactions and influence disease outcomes.

## 4 Materials and methods

### 4.1 Mtb culture

MtbHN878 was grown at 37 °C with 5% CO_2_, to mid-log phase (OD600 ~0.6) in Middlebrook 7H9 broth (Difco, MI, USA), supplemented with 0.5% glycerol, 0.25% Tween 80, and 10% OADC enrichment (BD Biosciences, MD, USA). Serial dilutions of culture were plated on Middlebrook 7H10 agar plates (Difco, MI, USA) and incubated at 37 °C with 5% CO_2_ supply for up to 6 weeks to enumerate the number of colony forming units (CFUs). An aliquot of stock culture was briefly sonicated to disrupt clumps, and either inoculated into fresh 7H9 media for total RNA isolation or diluted to 1x10^6^ CFU/ml in sterile saline and used for rabbit aerosol infection, as reported (16, 60).

### 4.2 Rabbit infection and histology

Female New Zealand White rabbits aged 10 to 12 weeks (n=3-4) of about 2.5kg body weight were exposed to Mtb aerosols through a “nose-only” delivery system (C-H Technologies, NJ, USA) to implant ~3log_10_ CFU in the lungs as reported (16, 60). At 4-weeks post infection (acute stage), animals were euthanized by procedures approved by the American Veterinary Medical Association (AVMA) guidelines (13–16). Briefly, rabbits were sedated by intramuscular injection with a cocktail of Ketamine (35mg/kg) and Xylazine (5mg/kg), followed by euthanasia with intravenous injection of Euthasol (390mg/ml) diluted in sterile saline. Death was confirmed by lack of heartbeat, pupil dilation, tail pinch (no motion). Following autopsy, the lungs were harvested for Mtb RNA isolation and for histologic sectioning using H&E method as we described (16, 60). The histology sections were analyzed on a Nikon Microphot-FX microscope, and the images were acquired using the NIS-Elements software (Nikon Instruments, NY, USA).

### 4.3 Mtb microarray

The experimental design is shown in **Figure 1A**. Total Mtb RNA was isolated from broth- or rabbit lungs (n=3) by the TRIZol method and used for microarray analysis as reported (16). To obtain sufficient Mtb mRNA, equal amounts of all total RNA (500ng) were amplified using the MessageAmp II-Bacteria RNA amplification kit (Ambion). Since this method generates antisense aRNA, a cDNA synthesis step was performed using SuperScript II kit (Thermoscientific, CA, USA), followed by Cy3 or Cy5 labeling using the BioPrime kit (Thermoscientific, CA, USA), as previously described (61). This amplification approach has been validated for differential gene expression studies in multiple organisms, including Mtb from host cells and patient sputum and blood samples (61–64). We also confirmed previously that gene expression profiles from amplified RNA closely matched those from unamplified RNA (64–66). Mtb microarray slides containing 70-mer oligonucleotides representing all annotated open reading frames of the Mtb genome were obtained from the Center for Applied Genomics (Public Health Research Institute, Newark, NJ). Labeled cDNA were purified using Microcon YM10 filters (Millipore) and hybridized to the arrays overnight. The experiment was performed in triplicate (biological) with one Cy3/Cy5 dye swaps (technical replicate) as previously reported (63, 64, 67). Arrays were scanned using an Axon 4000B scanner and analyzed with GenePix Pro 6.1 software. Data were normalized using the print-tip Lowess method, and background correction. The Cy5/Cy3 expression ratios were calculated for each gene (63, 64, 67). Differential gene expression of Mtb genes in the lungs, relative to 7H9 broth, were analyzed using one-class Significance Analysis of Microarrays (SAM) in MultiExperiment Viewer (TMEV) (63, 64, 67). Genes exhibiting at least a 1.5-fold change with a false discovery rate (FDR) of 5% were considered statistically significant. The microarray data were deposited to the NCBI’s Gene Expression Omnibus (GEO; accession number: GSE326996).

### 4.4 Gene Ontology (GO) enrichment analysis

GO analysis was performed using *clusterProfiler* (v4.10.0) with Mtb-specific annotation database (68). The average log_2_ fold-change values across biological replicates were used as input, and genes with missing values across all replicates were excluded from analysis (68). A customized background gene set was employed to improve biological specificity by restricting enrichment testing to Mtb annotations (69) The organism-specific annotation package was constructed using *AnnotationForge* (v1.44.0). Mtb gene annotations were obtained from NCBI Gene (https://ftp.ncbi.nlm.nih.gov/gene/DATA/), and identifier mapping files from UniProt/ExPASy (https://ftp.expasy.org/databases/uniprot/current_release/knowledgebase/idmapping/). These datasets were integrated using the makeOrgPackageFromNCBI () function to generate the org.MtuberculosisH37Rv.eg.db package, which was used for all downstream analyses (70).

GO enrichment was conducted for biological process and molecular function ontologies using default parameters. Multiple testing correction was applied using the Benjamini–Hochberg method, and enriched terms were considered significant at p < 0.05 and FDR q < 0.05. Kyoto Encyclopedia of Genes and Genomes (KEGG) pathway enrichment analysis was performed in parallel using the same gene sets. Enriched GO terms were visualized using dotplot() from the *enrichplot* package, with each dot denoting an enriched term, where dot size representing gene counts and color indicating adjusted p-values (71). KEGG pathways were visualized using *Pathview* R package (72). Functional relationships among enriched terms were examined using enrichment map analysis via the emapplot() function in *enrichplot*. Pairwise semantic similarities were computed using pairwise_termsim(), and resulting networks were used to identify clusters of functionally related terms based on shared gene membership or semantic similarity (71).

### 4.5 qPCR analysis

Total Mtb RNA was converted to cDNA using a Sprint RT system (Clontech, CA) and used in qPCR as described (16, 17). Briefly, 10 nanograms of cDNA were mixed with Mtb gene-specific forward and reverse primers and 2xSYBR green master mix (Takara, CA). Mtb 16s rRNA gene was used as house-keeping control in all tested samples (**Table 1**). The qPCR was performed in a AriaMx Real-Time PCR System, and the data was analyzed using MxPro Software (Agilent Technologies, CA). Gene expression was calculated using the formula 2 ^−ΔCt^.

### 4.6 Statistical analysis

All experiments were performed with three biological replicate samples. Microarray was done with one additional dye-swap sample as control for probe-labeling. qPCR was repeated at least by two technical replicates using three biological samples. The data was analyzed using GraphPad Prism 10 (GraphPad Prism, USA). Pairwise analysis was performed using Students’ t-test, and for multiple comparison analysis, one-way-ANOVA with Tukey’s correction method was used. For all experiments, p-value ≤ 0.05 was considered statistically significant.

## Data Availability

The microarray data for Mtb grown in broth culture or from rabbit lungs, is deposited to the NCBI’s Gene Expression Omnibus (GEO) platform (Accession number: GSE326996). Analyzed data is presented in the **Supplementary Files**. The data used in the tables and figures are available from the corresponding author upon request.

## Supplementary material

Supplementary Figure 1 KEGG map of Mtb polyketide biosynthesis pathway in infected rabbit lungs. Upregulated genes are indicated in red color, and downregulated genes are in green color.

Supplementary Figure 2 KEGG map of Mtb mycolic acid biosynthesis pathway in infected rabbit lungs. Upregulated genes are indicated in red color, and downregulated genes are in green color.

Supplementary Table 1 Differentially expressed Mtb genes in the lungs with a 3-fold cutoff.

Supplementary Table 2 GO terms, gene IDs, expression levels, and p-values of differentially regulated Mtb biological processes in the lungs.

Supplementary Table 3 GO terms, gene IDs, expression levels, and p-values of differentially regulated Mtb molecular functions in the lungs.
